# Direct evidence for grain boundary passivation in Cu(In,Ga)Se_2_ solar cells through alkali-fluoride post-deposition treatments

**DOI:** 10.1038/s41467-019-11996-y

**Published:** 2019-09-04

**Authors:** Nicoleta Nicoara, Roby Manaligod, Philip Jackson, Dimitrios Hariskos, Wolfram Witte, Giovanna Sozzi, Roberto Menozzi, Sascha Sadewasser

**Affiliations:** 1 International Iberian Nanotechnology Laboratory (INL), Av. Mestre José Veiga s/n, 4715-330 Braga, Portugal; 2 Zentrum für Sonnenenergie- und Wasserstoff-Forschung Baden-Württemberg (ZSW), Meitnerstr. 1, 70563 Stuttgart, Germany; 3 Department of Engineering and Architecture, University of Parma, Parco Area delle Scienze 181A, 43124 Parma, Italy

**Keywords:** Solar cells, Solar cells, Electronic properties and materials

## Abstract

The properties and performance of polycrystalline materials depend critically on the properties of their grain boundaries. Polycrystalline photovoltaic materials – e.g. hybrid halide perovskites, copper indium gallium diselenide (CIGSe) and cadmium telluride – have already demonstrated high efficiencies and promise cost-effective electricity supply. For CIGSe-based solar cells, an efficiency above 23% has recently been achieved using an alkali-fluoride post-deposition treatment; however, its full impact and functional principle are not yet fully understood. Here, we show direct evidence for the passivation of grain boundaries in CIGSe treated with three different alkali-fluorides through a detailed study of the nanoscale optoelectronic properties. We determine a correlation of the surface potential change at grain boundaries with the open-circuit voltage, which is supported by numerical simulations. Our results suggest that heavier alkali elements might lead to better passivation by reducing the density of charged defects and increasing the formation of secondary phases at grain boundaries.

## Abstract (web summary)

Grain boundaries play critical roles in determining the properties and performance of solar cells based on polycrystalline materials. Here Nicoara *et al*. showcase that proper treatments passivate defects at grain boundaries by forming secondary material phases with the CIGSe absorbers and lead to higher Voc.

## Introduction

The use of polycrystalline materials for photovoltaic (PV) energy conversion promises fast fabrication and high cost-savings potential. However, the properties of polycrystalline semiconductors are frequently dominated by the properties of grain boundaries (GBs). In recent years, thin-film solar cells based on polycrystalline cadmium telluride (CdTe), Cu(In,Ga)Se_2_ (CIGSe), and lead-halide perovskite absorbers have surpassed the barrier of 22% power conversion efficiency^1^. These outstanding efficiencies have been achieved through tedious materials and device optimization. It is obvious that in such high-efficiency devices the GBs are not leading to strong carrier recombination^2^. In fact, some of the strategies for efficiency improvement have subsequently been identified to passivate GBs, e.g. a cadmium-chloride (CdCl_2_) treatment in CdTe solar cells^3–5^ and a polymer treatment in perovskite solar cells^6–9^. For CIGSe solar cells an alkali-fluoride (AlkF) post-deposition treatment (PDT)^10,11^ has recently led to a significant increase in the efficiencies^12–15^. Indeed, it has been reported that for a potassium-fluoride (KF)-PDT the electronic properties of the GBs are beneficially modified^16^. However, a full understanding of the role of GBs in CIGSe is still lacking, especially in view of the heavier AlkF-PDT using rubidium-fluoride (RbF) and cesium-fluoride (CsF), which have led to higher record efficiencies in the last 2 years^13,15^.

Most of the studies investigating the role of GBs in CIGSe were performed on material without any AlkF-PDT^17–26^. The only alkali elements present in the absorber were those diffusing from the soda-lime glass substrate, mainly sodium (Na). Compositional studies at atomic level using atom probe tomography (APT) indicated Na accumulation at the GBs^27,28^. More recent studies, where a KF-PDT was applied indicate also accumulation of K at GBs^29,30^. As a consequence of the relative concentrations of the different alkali elements at GBs and in the grain interior, several effects have been observed^31^, including Cu depletion, In and Se accumulation at GBs^32^, a diminished downward^33^ and increased upward^16^ band bending at GBs, increase^34–36^ or decrease^10^ of carrier concentration, and an impact on the GB recombination velocity^37^. A similar range of observations has been reported for RbF-PDT^38–41^. The detailed composition, chemistry, and microstructure at GBs leads to different GB and possibly also bulk properties, explaining discrepancies in the literature^42^. Different models have been suggested to explain the properties of GBs, however, there is no agreement about the relevance of the electronic properties of GBs for the device performance.

We present a comprehensive Kelvin probe force microscopy (KPFM) study of the electronic GB properties in CIGSe deposited by co-evaporation and compare the effect of KF-, RbF-, and CsF-PDT. A statistical analysis of more than 240 GBs shows distinct differences of the potential variation across the GBs between the samples. To isolate the effect of the different AlkF-PDTs, we use nominally identical CIGSe absorbers grown with the same processing conditions. To understand the impact of GBs on the device performance, we perform three-dimensional (3D) device simulations considering different GB electronic properties. Our results indicate that GBs exhibiting downward band bending (i.e. a hole transport barrier) have a negative impact on device performance. We also find that an optimized AlkF-PDT can lead to a passivation effect at GBs.

## Results

### Kelvin probe force microscopy

**Fig. 1:**
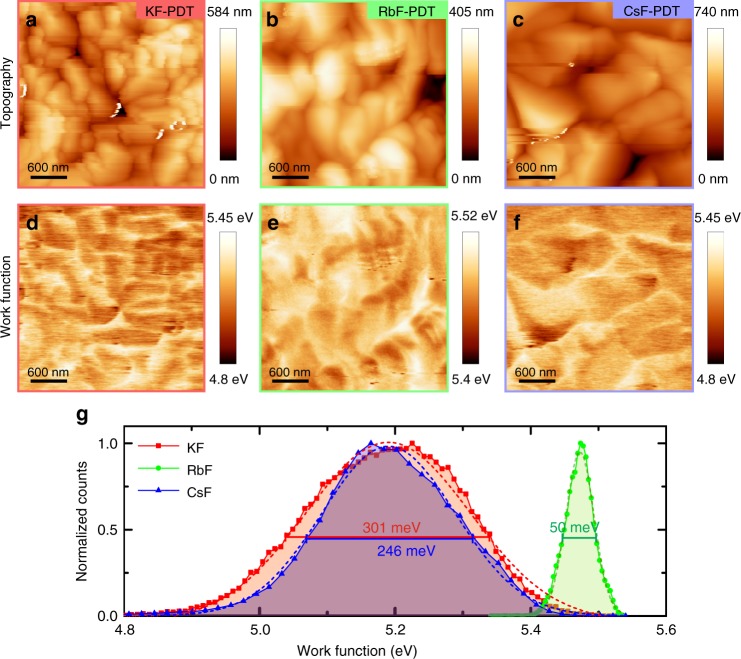
Representative KPFM results on annealed and rinsed AlkF-PDT CIGSe absorbers. From left to right the data correspond to KF-, RbF-, and CsF-PDT. **a**–**c** Topography images, **d**–**f** simultaneously acquired work function maps measured under dark conditions, and **g** histograms extracted from the work function maps; dashed lines indicate Gaussian fits and the bars with the numbers the spread at 1/*e* of the peak maximum

The CIGSe absorbers with the different AlkF-PDTs (Alk = K, Rb, and Cs) were examined by KPFM. To obtain reliable quantitative information, simultaneous measurements of topography and contact potential difference (CPD) were taken at different sample locations, separated by several hundred µm with a total of 20–50 measurements per sample. This approach ensures a statistically relevant characterization of the surface potential. The general surface morphology (Fig. 1a–c), is independent of the alkali element used for the PDT, exhibiting grains with typical size of ~1 μm and with smooth facets. Simultaneously acquired work function maps (Fig. 1d–f) from the same areas as the topography images show a significant variation depending on the alkali element, as illustrated by the respective histograms in Fig. 1g. From the histograms, the peak maximum and the spread of the potential at 1/*e* of the maximum were analyzed. The average values of peak maximum and spread from all inspected areas show a single, narrow distribution for RbF and two separated distributions for KF and CsF (see Supplementary Fig. 1 and Supplementary Note 1). We do not observe a specific trend of the work function from lighter to heavier alkali elements. However, we clearly observe that the RbF-PDT has a more homogeneous work function distribution than the KF- and CsF-treated CIGSe. We note that the CIGSe deposition and PDT were optimized for RbF and that the CIGSe growth was maintained identical between the different AlkF-PDTs to permit discerning the effect of the alkali elements on the absorber and device properties.

**Fig. 2:**
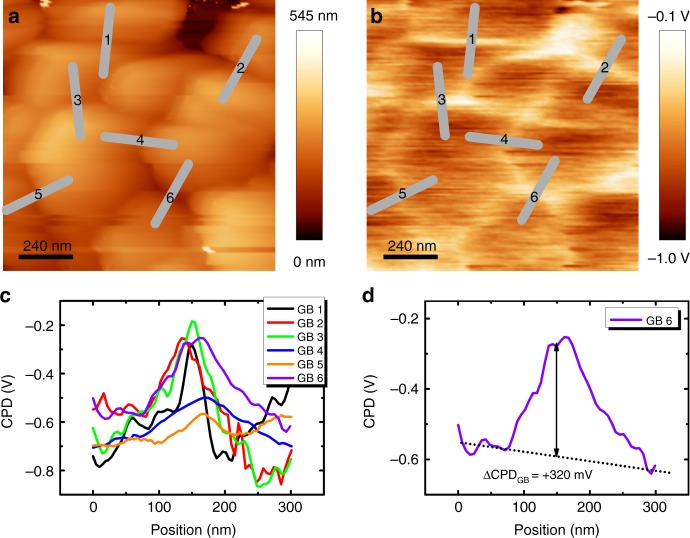
KPFM measurements on KF-PDT CIGSe. **a** Topography and **b** simultaneously acquired contact potential difference (CPD) map in dark conditions. The GBs analyzed in this study are marked by grey lines that cross the GB perpendicular. **c** Line profiles extracted from the CPD image. **d** Individual line profile of GB 6 to illustrate that the potential variation (ΔCPD_GB_) of each GB corresponds to the difference between CPD_GB_ at the GB and the average < CPD > _GS_ obtained on the grain surfaces of the adjacent grains

**Fig. 3:**
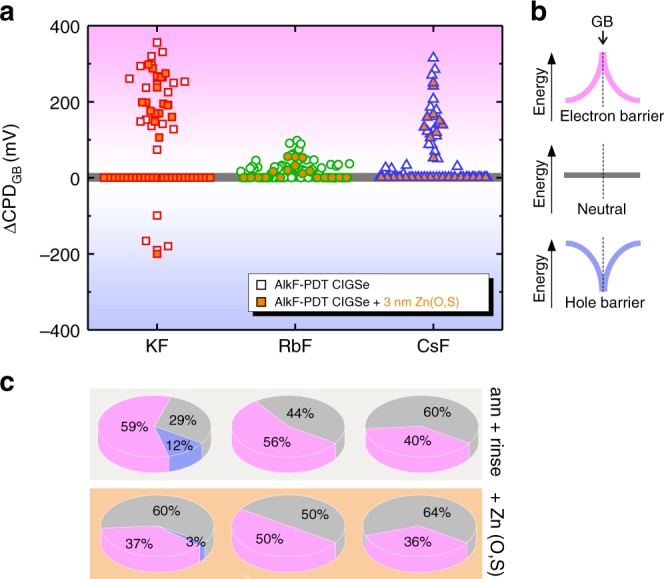
Potential variations at grain boundaries. **a** Contact potential difference at GBs (ΔCPD_GB_) extracted from KPFM measurements in the dark on KF- (open red squares), RbF- (open green circles), and CsF-PDT CIGSe (open blue triangle). The orange-filled symbols correspond to identical samples with a Zn(O,S) buffer layer from an 8 min. CBD process. **b** Schematic potential profiles for the different types of GBs: negative ΔCPD_GB_ or dip-like shape (blue), negligible ΔCPD_GB_ or neutral (grey) and positive ΔCPD_GB_ or spike-like shape (pink) and the corresponding models (hole or electron barrier). **c** Pie-charts indicating the proportion of the GB types found in CIGSe with different AlkF-PDT (top row) and on identical samples with Zn(O,S) layer (bottom row)

To further investigate the work function variations between the different AlkF-PDTs, the locally-induced effect on the electronic properties of the GBs was analyzed. For all samples, the identification of GBs was easily possible from the topography images (Fig. 1a–c). The CPD profile across GBs was then extracted from the simultaneously acquired CPD maps^16,21^, as illustrated in Fig. 2 and detailed in the Supplementary Note 3. The CPD variation across the GBs (ΔCPD_GB_) is then determined from individual line profiles (see Fig. 2d). It was previously shown that the identification of GBs based on topography images leads to the predominant selection of GBs with random orientation, with only a small fraction of identified symmetric (Σ3) GBs^22,43^. In fact, it has been widely shown that symmetric GBs are electronically inactive and appear to be benign for solar cell performance^17,18,44^. We performed a statistical analysis of 20–65GBs per sample, measured at different positions on the sample. The results of ΔCPD_GB_ for all investigated samples are plotted in Fig. 3a. It is noted that in the present study the CPD is proportional to the energy. Therefore, a positive potential difference at the GB means a higher potential at the GB as compared to the grain surface (GS), which corresponds to an electron barrier (Fig. 3b). Conversely, a negative potential difference at a GB represents a hole barrier. Whenever the potential variation across the GB is very small with respect to contiguous grains, the GB is considered neutral (ΔCPD_GB_ = 0).

The distributions of the occurrence of different GB types exhibit some differences between the three AlkF-PDTs (Fig. 3c). KF-treated CIGSe (Fig. 3a, open red squares) shows the widest distribution of the potential variation across GBs, with almost 60% of the GBs exhibiting an electron barrier and 12% of the GBs exhibiting a hole barrier; the remaining 29% are neutral GBs. The magnitude for both electron and hole barriers varies by approximately 100 mV around ±200 mV. RbF-CIGSe (open green circles) shows a significantly different distribution of GB types, with an almost 50:50 ratio between GBs without a barrier and GBs with an electron barrier, and a ΔCPD_GB_ magnitude with values below 100 mV. Similarly, CsF-CIGSe (open blue triangles) presents also about a 50:50 ratio between electron barriers and GBs without a barrier; however, the magnitude of the electron barrier is larger with a variation of ~100 mV around ΔCPD_GB_ ≈ 200 mV. Importantly, in contrast to the KF sample, RbF- and CsF-treated CIGSe do not show any GBs with negative potential difference (hole barrier).

To confirm the validity of the above KPFM results at the GBs and ensure that the studied sample surface is representative for the solar cell device pn-junction, we also investigated samples coated with a thin zinc oxysulfide (Zn(O,S)) buffer layer grown by chemical bath deposition (CBD) immediately after CIGSe deposition. We chose an 8 min deposition time to grow an ~3 nm thick layer onto the three alkali-treated CIGSe surfaces to ensure that KPFM observation of the GBs in CIGSe is still possible. The thin Zn(O,S) layer only slightly modifies the work function of the samples (Supplementary Fig. 1 and Supplementary Note 2) in agreement with previous results on a cadmium sulfide (CdS) buffer layer, and indicating that 3 nm of Zn(O,S) buffer is insufficient to form a pn-junction^16,45^. The electronic GB properties of the Zn(O,S)-coated CIGSe samples with the three AlkF-PDTs (orange-filled symbols in Fig. 3a) are very similar to those observed for the CIGSe samples after annealing and rinsing (i.e. without any buffer layer deposition). The most significant change is observed for KF-CIGSe/Zn(O,S), where a larger fraction of GBs without a barrier appears. Otherwise, the magnitude and distribution of GB types are similar between the CIGSe surface and the Zn(O,S)-coated CIGSe surface, confirming that the observed GB properties are reflecting the properties of the CIGSe material close to the pn-junction as present in the full device.

### Solar cell device simulations

**Fig. 4:**
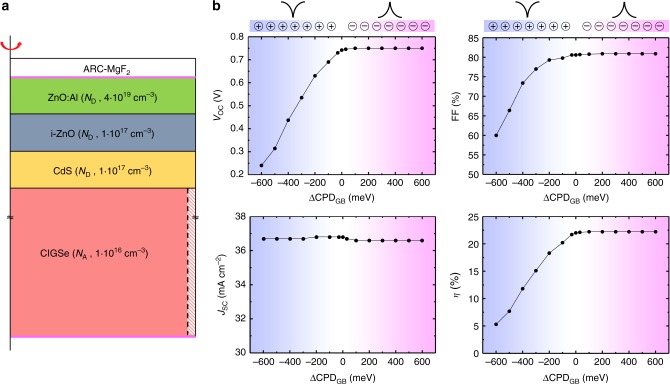
Numerical simulations of solar cell devices with charged grain boundaries. **a** Simulated structure and **b** solar cell figures of merit as a function of band bending at the GB. ΔCPD_GB_ < 0 (i.e. positive fixed charge at the GB) leads to downwards band bending, while ΔCPD_GB_ > 0 (i.e. negative fixed charge at the GB) leads to upwards band bending. Strong losses in *V*_oc_, FF, and *η* are observed for increasing downward band bending

To understand the impact of the observed GB potential profiles on the figures of merit of respective solar cell devices, we performed numerical simulations based on the Synopsys Sentaurus TCAD suite^46^, using a three-dimensional cylindrical model of a grain surrounded by a GB (Fig. 4, see further details in the Supplementary Note 4). To get clear qualitative indications on the effect of GB band bending on the cell performance, and in the absence of detailed experimental data on the electrical features of GB defects, here we simulated the GB as a thin (1 nm) region surrounding the grain, decorated with fixed charge (some indications about the effects of decorating the GB with electrically active traps, as opposed to fixed charge, can be found in the Supplementary Note 4). In the absence of the GB the simulated cell exhibits an open-circuit voltage *V*_OC_ = 742 mV, short-circuit current density *J*_SC_ = 36.8 mA cm^−2^, fill factor FF = 80.6%, and power conversion efficiency *η* = 22%. Placing fixed charge *Q*_f_ at the GB allows to simulate the band bending observed by KPFM: the GB is thus decorated either by fixed positive or fixed negative charges, inducing downward or upward band bending (ΔCPD_GB_), respectively. The charge density *Q*_f_/*q* at the GB is varied in the range from −1.25 × 10^14^ cm^−2^ to +2 × 10^11^ cm^−2^ to obtain ΔCPD_GB_ values in the dark between 600 mV and −600 mV, respectively.

The results of this simulation (Fig. 4) clearly show qualitatively different behavior between positively and negatively charged GBs. Positively charged GBs (i.e. GBs with a downward band bending, corresponding to a hole barrier) severely affect the solar cell performance, leading to strong losses in *V*_oc_ and FF and, consequently, in efficiency; the larger the negative ΔCPD_GB_, the lower are *V*_oc_, FF, and *η*. This remarkable degradation of performance is due to increased electron density at the GB, leading to the formation of an electron channel (Supplementary Fig. 4). As a result, the junction is shunted, more and more as the downward band bending gets stronger, as clearly shown by a shift in the dark current-voltage (*J*-*V)* curves as ΔCPD_GB_ becomes more negative (Supplementary Fig. 5a), in qualitative agreement with the experimental data (Supplementary Fig. 5c). Nevertheless, efficiencies *η *≥ 20% are compatible with negative ΔCPD_GB_ less than −100 mV, corresponding to positive charge densities at the GB smaller than 10^11^ cm^−2^. On the other hand, negatively charged GBs (i.e. GBs with a positive ΔCPD_GB_, corresponding to an electron barrier) have negligible impact on the performance parameters of the solar cell. In this case, the upward band bending repels electrons from – and accumulates holes at – the GB, which does not entail any junction shunting and has negligible effects on the *J*-*V* curve (Supplementary Fig. 5b); it is also worth pointing out that the simple fixed-charge GB model used here does not include possible effects of enhanced non-radiative recombination at the GB (see Supplementary Note 4 for a more detailed discussion of this point).

## Discussion

The work function measured in KPFM depends on individual or a combination of the following contributions: i) the measured material through its electron affinity and surface dipole and ii) the charge density or the presence of fixed charges. These effects apply to the work function measurement on the surface, as well as at the GBs.

The electronic properties of alkali-treated samples can be related to the alkali incorporation into the CIGSe. The obtained values of the overall work function show small differences between the different alkali elements used for the PDT (Supplementary Fig. 1), where a slight correlation to the open-circuit voltage of respective devices is observed (Supplementary Fig. 2). This correlation could indicate that the observed change in work function partially results from a change in the doping concentration of the CIGSe absorber and consequently a shift in the Fermi-level^34,47^. However, we cannot exclude that the different alkali elements lead to the formation of different surface phases. For CIGSe subjected to a KF-PDT, KInSe_2_ and In_2_Se_3_ surface phases were reported leading to a band gap widening and to changes in the conduction band edge^48–50^. For RbF-PDT samples the occurrence of a similar surface phase is still under debate^51–54^.

Nevertheless, our main observations relate to the GBs in CIGSe with AlkF-PDT (as shown in Fig. 3) and can be summarized as follows: (i) all studied samples except for the annealed-rinsed KF-PDT sample show about half of the GBs with no potential variation (GBs with no barrier). (ii) All samples show GBs with electron barriers, while (iii) only the KF-PDT sample shows a non-negligible fraction of GBs with hole barriers. (iv) The magnitude of the potential barriers at the GBs found for RbF-PDT is significantly smaller than that found for KF- and CsF-PDT. As stated above, the CPD variation at the GBs can result from a change in the material or the presence of localized charges.

Recent studies imaging the atomic-scale elemental distribution at and around GBs in CIGSe by atom probe tomography (APT) and by nano X-ray fluorescence (nano-XRF) have shown a segregation of the heavy alkali elements for RbF- and KF-PDT^38,39,55^. At the same time, the lighter Na (which diffuses from the soda-lime glass during CIGSe deposition at elevated temperatures) is displaced into the surrounding CIGSe grain interiors, where it is assumed to modify the doping concentration^34,41,47^. The concentration of the alkali elements at the GBs ranges between 0.1 and 3.7% (Supplementary Table 2), as determined by APT and transmission electron microscopy (TEM) studies^26–30^, and even up to 24%, determined by nano-XRF^39^. On the other hand, density function theory (DFT) has demonstrated that the formation of an AlkInSe_2_ phase is energetically favorable over the mixed (Alk,Cu)InSe_2_ phase for the heavier alkali elements K, Rb, and Cs^56^. Furthermore, the thermodynamic stability range of the AlkInSe_2_ phase in the phase diagrams increases from K → Cs (Supplementary Fig. 7). Therefore, with the concentration of the heavy alkali elements of a few atom percent, the formation of secondary phases of AlkInSe_2_ becomes highly probable. On the other hand, the presence of charged defects has long been argued to be responsible for CPD variations at GBs as observed by KPFM^16,19,57^. We note here that in our previous study^16^ of KF-PDT CIGSe the observed ΔCPD_GB_ values were smaller than those observed on the present KF-PDT samples, but rather similar to those observed here for the RbF-PDT sample. While this similarity might reflect that the PDT in our previous study was optimized for KF-PDT, we cannot exclude that the smaller values observed in that study are due to a larger tip-sample distance, typical for measurements in air-KPFM^58^.

With the goal to discern between these two scenarios (secondary phase vs. charges at GBs), we also measured the surface photovoltage (SPV) at the GBs by analyzing additional KPFM data taken under illuminated conditions (here we define SPV = CPD_light_ - CPD_dark_). In case of the presence of a different material at the GB, no change upon illumination is expected, while photo-generated charges are expected to change the charge state at the GBs, leading to a modification of the CPD and thus the observation of a SPV^59^. The statistical analysis of the SPV at the GBs for the annealed-rinsed CIGSe samples subjected to the different AlkF-PDTs (Supplementary Fig. 8 and Supplementary Note 5) indicates an increasing fraction of uncharged GBs toward heavier alkali elements (the CsF-PDT sample has more than 70% of the GBs uncharged). At the same time, a decreasing fraction of charged GBs toward heavier alkali elements is observed, and prominently, a decreased fraction of positively charged GBs, which were identified to be detrimental to solar cell performance in the simulations presented above. Assuming that the photo-excited charges screen the fixed charges at the GBs, the ΔCPD_GB,illum_. under illumination should be related to a band offset at the GB (see Supplementary Fig. 9) with values of (221 ± 73) meV for KF-PDF, (40 ± 23) meV for RbF-PDF, and (108 ± 21) meV for CsF-PDF. These values are in the range of the variation of the band gap between the respective AlkInSe_2_ compounds^56^. The lowest value observed for the RbF-PDT sample might reflect that the RbF-PDT and CIGSe growth were optimized with respect to each other for best performance of the solar cell devices. Nevertheless, the large fraction of uncharged GBs for the CsF-PDT could indicate that further optimization might lead to improved performance also for a CsF-PDT; in fact, the former world record CIGSe solar cell with 22.9% efficiency was obtained using a CsF-PDT process^14^.

We also explored the effect of a secondary phase – i.e., AlkInSe_2_ compound – at the GB by numerical simulations. The 1 nm thick GB region surrounding the grain (see Fig. 4a) has been modeled in this case as a different material, with bandgap of 2.53 eV^56^; to cover the range of conduction band offsets discussed above – i.e., 221, 40, and 108 meV for KF-PDF, RbF-PDF, and CsF-PDF, respectively – we simulated different conduction band offsets (electron barriers) of 400, 200, and 50 meV, as well as the case where the conduction band offset is zero and the valence band offset (hole barrier) equals the whole bandgap difference between the CIGSe and the secondary phase. Consistently with the simulation result shown in Fig. 4b, where no effect is seen for electron barriers at the GB, the cell’s performance is not in the least modified by the presence of the secondary phase at the GB. In the presence of GB defects – as discussed above and also shown for the case where the secondary phase is present at the CIGSe surface^60^ – the higher bandgap AlkInSe_2_ compound is expected to provide partial or complete defect passivation, depending on the specific defect characteristics.

**Fig. 5:**
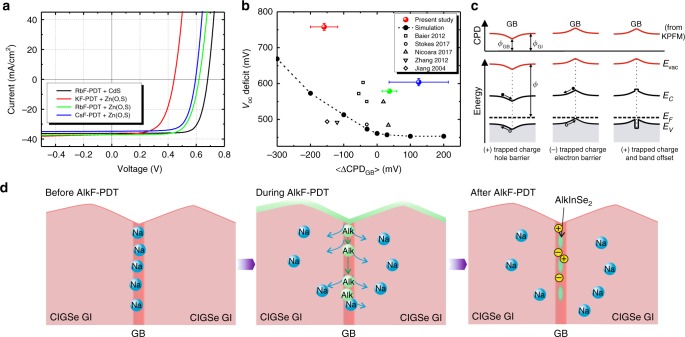
Role of grain boundaries in solar cell devices. **a** *J*–*V* characteristics of reference solar cells for the CIGSe layers with different AlkF-PDT. **b** Open-circuit voltage deficit with respect to the respective band gap as a function of the potential change at the GBs for the three studied samples (large colored circles) and for some results taken from the literature^16,20,21,55,63^. The black dashed curve shows the results obtained from our simulations. **c** Schematic band diagrams (lower panels) and expected CPD as measured by KPFM (upper panels) for a GB with a hole barrier (left), an electron barrier (middle) and an electron barrier with a band offset (right). **d** Schematic of the PDT process illustrating the displacement of Na to the grain interior by the heavier alkali-elements used in the PDT and the possible formation of secondary phases and charged defects at the GB

Figure 5 presents the overall results of our study, relating the observed GB properties to the solar cell device properties and illustrates the proposed action of the heavy-alkali fluoride PDTs. The *V*_oc_ analyzed from the *J*–*V* curves (Fig. 5a) of the studied reference devices with CBD-Zn(O,S) buffer layers shows a clear dependence on the potential change observed at the GBs (Fig. 5b), which is explained by our numerical simulations (black dashed curve). It should be noted that the *J*-*V*-curves were measured in the as-grown state and *V*_oc_ values of Zn(O,S)-buffered devices could be significantly improved after a light-soaking and/or a post-annealing procedure. For comparison, also some values from the literature are included. We therefore propose the following processes during the PDT: initially, a high concentration of Na which has diffused from the soda-lime glass is found at the GBs. During the PDT, the heavier alkali elements diffuse into the grain boundaries and displace the lighter Na into the grain interior, where they occupy Cu vacancies and modify the charge-carrier concentration^34^. The heavier alkalis can lead to the partial formation of AlkInSe_2_ phases at the GB^56^, nevertheless, some charged defects remain at the GBs. The AlkInSe_2_ phase leads to a band offset due to the larger band gap and a reduction of charged defects (passivation effect). Nevertheless, in the studied samples some charged defects still remain. Our results indicate that the CsInSe_2_ formation might act more efficiently for the passivation of charged GB defects, in agreement with the lower formation enthalpy of this compound over the other AlkInSe_2_ phases^56^. However, obtaining an efficient passivation of the GBs through the AlkF-PDT requires a careful fine-tuning of the CIGSe deposition in combination with the PDT conditions. In fact, Malitckaya et al.^56^ show that the stability region of the AlkInSe_2_ phases depends on the detailed Cu, In, and Se chemical potentials, and that CsInSe_2_ shows the largest stability region (Supplementary Fig. 7). On the other hand, we attribute the low performance of the KF-PDT sample to a non-optimized combination of CIGSe deposition and KF-PDT process. Our results strongly suggest that GB passivation in this sample did not function properly and the observed inferior performance can be (at least partially) ascribed to non-passivated GBs. Specifically, when the figure of merit (*V*_oc_) has ‘good values’, the GBs have a low potential barrier, and vice versa, a large GB downward band bending has a detrimental influence on the device performance through losses in *V*_oc_ and FF.

In conclusion, using Kelvin probe force microscopy imaging, solar cell device characteristics, and numerical 3D device simulations, we could correlate the detrimental effect of downward band bending at grain boundaries in CIGSe absorbers with losses in the device performance of respective thin-film solar cells, predominantly with losses in the open-circuit voltage. Alkali-fluoride post-deposition treatments can passivate charged defects at grain boundaries and lead to the formation of alkali-indium-selenide phases which form more likely for heavier alkali elements K, Rb, and Cs. Our findings indicate that a careful optimization of the alkali-fluoride post-deposition treatment in conjunction with the CIGSe growth process is required to achieve efficient grain boundary passivation, likely controlled by the thermodynamic balance for the AlkInSe_2_ phase formation. The direct observation of such phases at grain boundaries will be the challenging task for future studies. Our findings are relevant in view of industrial production of full-size CIGSe solar modules, since for slight variations in the CIGSe material, alkali-fluoride PDTs can effectively passivate charges at grain boundaries and therefore mitigate losses due to the presence of GBs. Therefore, AlkF-PDT is an interesting option for industrial fabrication to make the material more homogeneous over large areas. Furthermore, the presented method to perform and analyze Kelvin probe force microscopy in dark and under illumination serves as a blueprint for the study of grain boundaries in other energy materials and polycrystalline materials in general.

## Methods

### Samples

CIGSe absorbers were deposited onto molybdenum (Mo)-coated alkali-aluminosilicate glass, which serves as a Na source during the three-stage evaporation process following the ZSW standard procedure established for the growth of high-efficiency CIGSe solar cells. CIGSe absorbers and the alkali-fluoride PDT were prepared in the same vacuum chamber without breaking the vacuum. Both CIGSe growth and AlkF-PDT were performed according to ref. ^13^. In the present study, the growth of the CIGSe was optimized for the RbF-PDT. Here “optimized” refers to a predictive correction for temperature and evaporation rate drifts based on long-term observation of these drifts, which requires a continuous observation of a series of processes. Naturally, switching from one alkali-fluoride post deposition treatment to another one does not allow such predictive correction due to a lack of long-term observation of drifts. The [Cu]/([In] + [Ga]) (CGI) and [Ga]/([In] + [Ga]) (GGI) ratios for the three CIGSe runs were determined by X-ray fluorescence measurements to 0.90 ± 0.01 and 0.32 ± 0.02, respectively. CIGSe from three consecutive and nominally identical runs was used to evaporate KF, RbF, and CsF onto the absorber surface. All analyzed samples were annealed and rinsed after the PDT process and before sending them for the KPFM measurements. In addition to these CIGSe surfaces, we also studied CIGSe onto which a thin Zn(O,S) buffer layer (thickness ~3 nm) was grown by an 8 min chemical bath deposition (CBD) process. Details of the CBD process are given in Ref. ^61^. For the reference solar cells a Zn(O,S) buffer layer with a thickness of 20 nm was grown by a 20 min CBD process. We note that respective reference solar cells with a CdS buffer layer reach efficiencies up to 20% (0.5 cm^2^ area with anti-reflective coating and after 15 min cold light soaking). Surface contamination during shipping was minimized by sealing all samples in N_2_ atmosphere directly after preparation. During mounting the samples on holders for the ultra-high vacuum (UHV) KPFM system (base pressure below 10^−10^ mbar), they were exposed for less than 10 min to air.

### Kelvin probe force microscopy

KPFM measurements on the CIGSe surface were carried out in a scanning probe microscope (Omicron Nanotechnology GmbH), controlled by a Nanonis controller (SPECS Zurich GmbH) using Pt/Ir-coated Si cantilevers (Nanosensors). Topography images were acquired using the frequency modulation technique at the fundamental resonance of the cantilever (frequency *f*_0_ ~ 165 kHz). Amplitude modulation (AM) KPFM was used for the detection of the contact potential difference (CPD) with an applied bias *V*_AC_ = 300 mV tuned to the second resonance frequency of the cantilever (*f*_2_ ~ 1.035 MHz). The CPD is the work function (*Φ*) difference between sample and tip: CPD = *Φ*_sample_−*Φ*_tip_. Reference measurements on Au surfaces were used to calibrate the work function of the tip (*Φ*_tip_) and to ensure comparability of the obtained CPD values. Subsequently, the work function of the sample was calculated from the CPD data according to *Φ*_sample_ = CPD + *Φ*_tip_. KPFM measurements under illumination were performed using a 635 nm wavelength laser with ~100 mW cm^−2^ power.

### Numerical simulations

Three-dimensional (3D) numerical simulations of the solar cell were performed with the Synopsys Sentaurus TCAD suite^46^ which calculates the charge-carrier distribution and transport by solving the Poisson, electron and hole continuity, and drift-diffusion equations. The Shockley–Read–Hall (SRH) model is included to account for non-radiative recombination. Light propagation is calculated by the transfer matrix method (TMM) under AM1.5 G solar spectrum illumination. The simulated cell features the standard stack made of Al-doped ZnO (AZO) and highly resistive i-ZnO window, CdS buffer, and CIGSe absorber with the double-graded GGI composition of the 21.7% efficiency cell reported in ref. ^12^; the measured GGI profile is loaded into the model to give the corresponding bandgap grading profile and complex refractive indices depending on both GGI and CGI ratios, as explained in ref. ^62^. The 3D model considers a cylindrical CIGSe grain with 1 μm diameter surrounded by a 1-nm-thick grain boundary; fixed charges of different density and polarity are considered in the GB to simulate the experimentally observed band bending.

### Reporting summary

Further information on research design is available in the Nature Research Reporting Summary linked to this article.

## Supplementary information

Supplementary Information

Solar Cells Reporting Summary

## Data Availability

The data that support the findings of this study are available from the corresponding author upon reasonable request.
