# Supplementary material for: Direct evidence for grain boundary passivation in Cu(In,Ga)Se2 solar cells through alkali-fluoride post-deposition treatments
Source: Nat Commun. 2019 Sep 4;10:3980. doi: 10.1038/s41467-019-11996-y (PMC6726603; doi:10.1038/s41467-019-11996-y)
Supplement: Supplementary file 1 — Supplementary Information [file 41467_2019_11996_MOESM1_ESM.pdf]

Supplementary Information for

Direct evidence for grain boundary passivation in $Cu(In,Ga)Se_2$ solar cells through alkali-fluoride post-deposition treatments

Nicoleta Nicoara[1], Roby Manaligod[1], Philip Jackson[2], Dimitrios Hariskos[2], Wolfram Witte[2], Giovanna Sozzi[3], Roberto Menozzi[3], and Sascha Sadewasser[1]

[1]International Iberian Nanotechnology Laboratory (INL), Av. Mestre José Veiga s/n, 4715-330 Braga, Portugal

[2]Zentrum für Sonnenenergie- und Wasserstoff-Forschung Baden-Württemberg (ZSW), Meitnerstr. 1, 70563 Stuttgart, Germany

[3]Department of Engineering and Architecture, University of Parma, Parco Area delle Scienze 181A, 43124 Parma, Italy

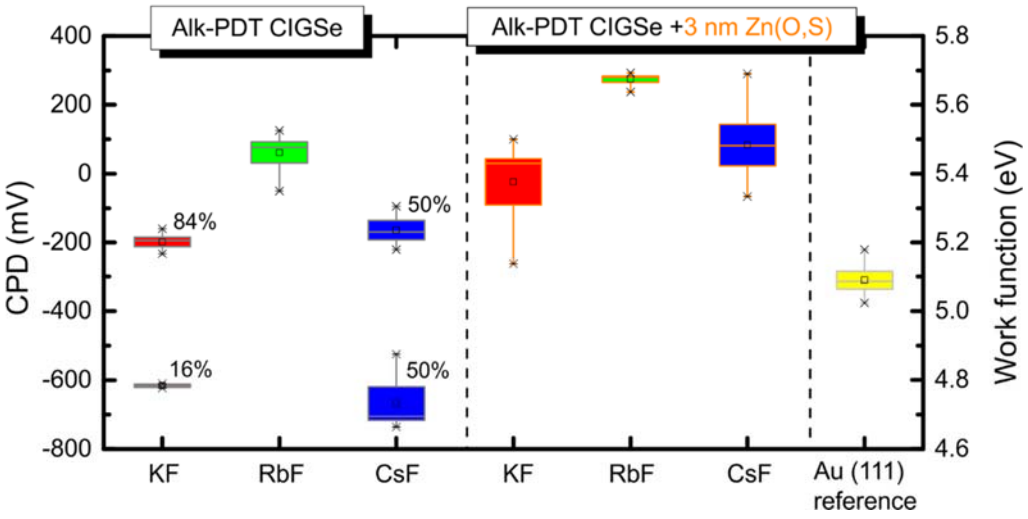

Supplementary Figure 1. KPFM statistical analysis of the surface potential of CIGSe as a function of the AlkF-PDT. Annealed and rinsed CIGSe absorbers and absorbers with an additional thin CBD-Zn(O,S) layer are compared. The percentage values give the relative distribution of the respective work function values. The values correspond to measurements taken under dark conditions.

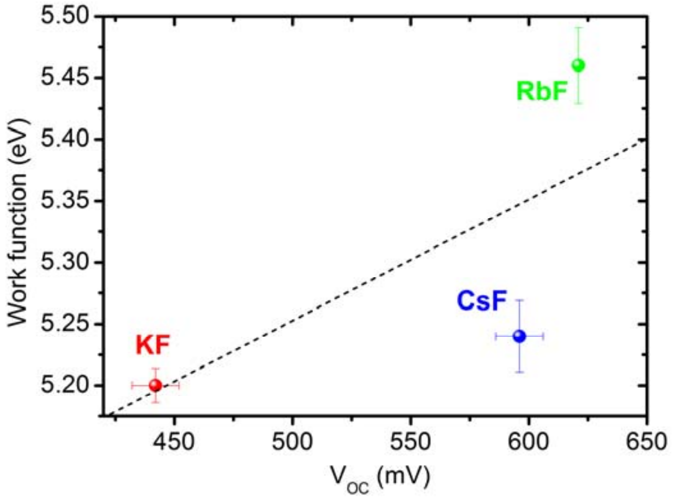

Supplementary Figure 2. Work function of the three AlkF-treated CIGSe samples vs. the open-circuit voltage of completed solar cell devices. The dashed line corresponds to a 1:1 ratio, indicating a change of 100 meV in work function for every 100 mV in V_{oc}. Horizontal error bars represent standard deviation and vertical error bars measurement uncertainty.

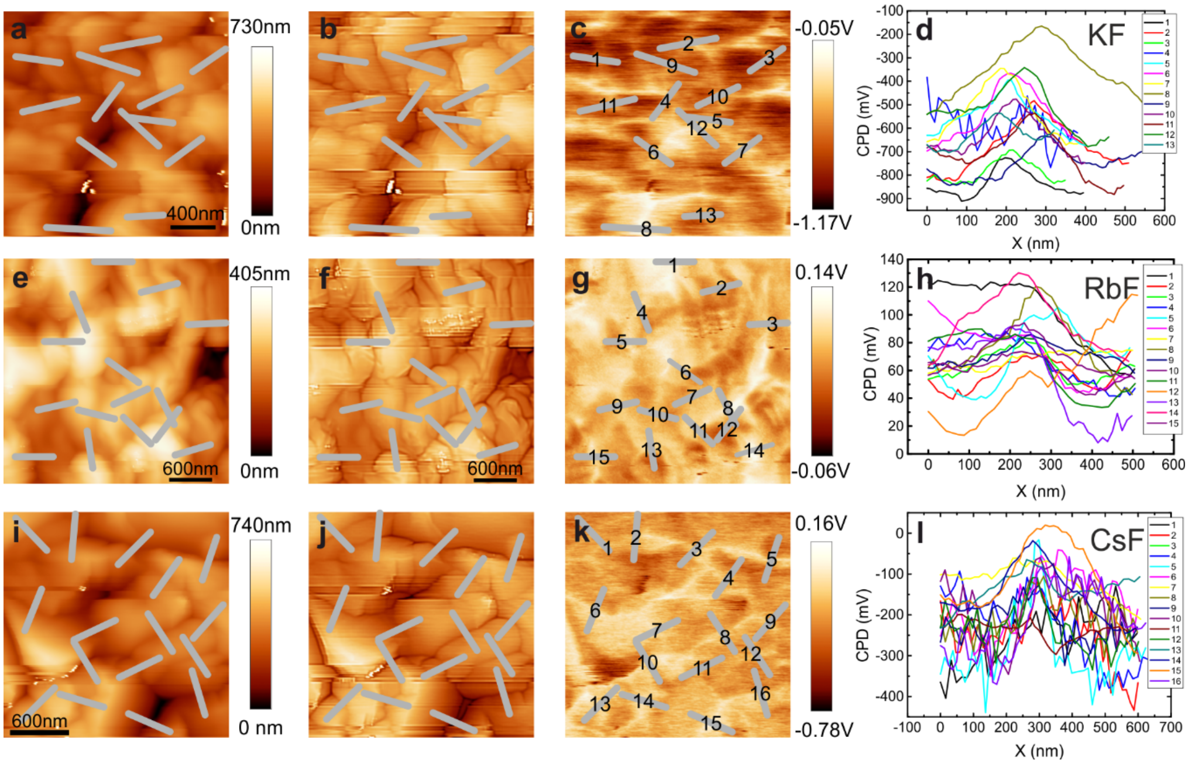

Supplementary Figure 3. KPFM measurements on the three AlkF-PDT CIGSe under dark conditions. **a**, **e**, **i** Topography and **c**, **g**, **k** simultaneously recorded contact potential difference (CPD) maps. Grey lines perpendicularly crossing GBs indicate the GBs selected for the analysis. **b**, **f**, **j** To ease GB identification, a Laplacian-transform was convoluted with the topography. **d**, **h**, **l** Profile lines across the GBs extracted from the CPD images. The potential variation (ΔCPD_{GB}) is extracted from these potential profiles as the difference between CPD_{GB} at the GB and the average $\langle CPD \rangle_{GS}$ on the grain surfaces (as shown in Fig. 2d of the main manuscript).

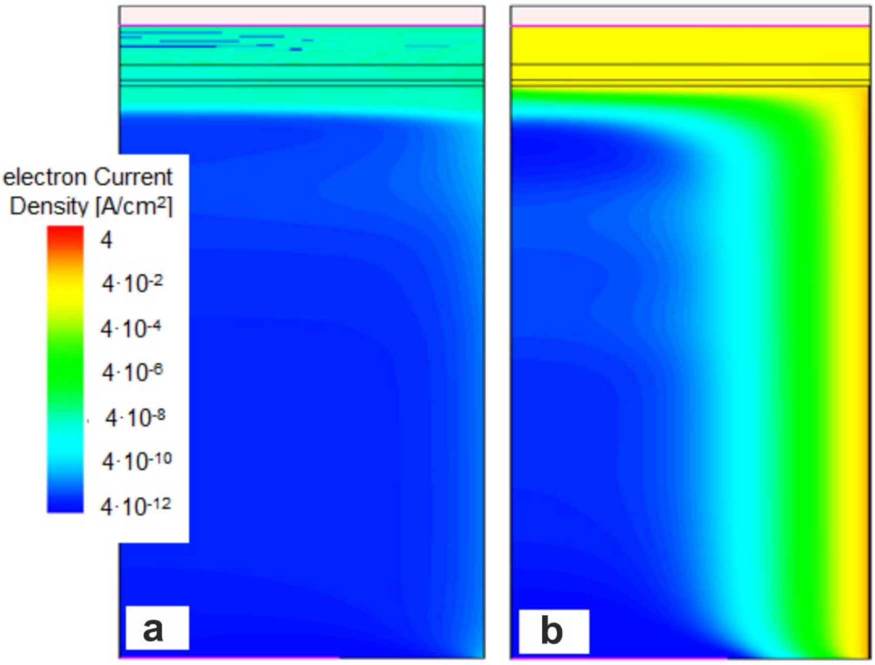

Supplementary Figure 4. Electron current density maps in the dark at a bias $V = 0.2$ V for **a** ΔCPD_{GB} = -30 mV (i.e., corresponding to a $Q_f/q = 5{\cdot}10^{10}$ cm^{-2} at the GB), and **b** ΔCPD_{GB} = -600 mV (i.e., corresponding to a $Q_f/q = 2{\cdot}10^{11}$ cm^{-2} at the GB). Figures not to scale.

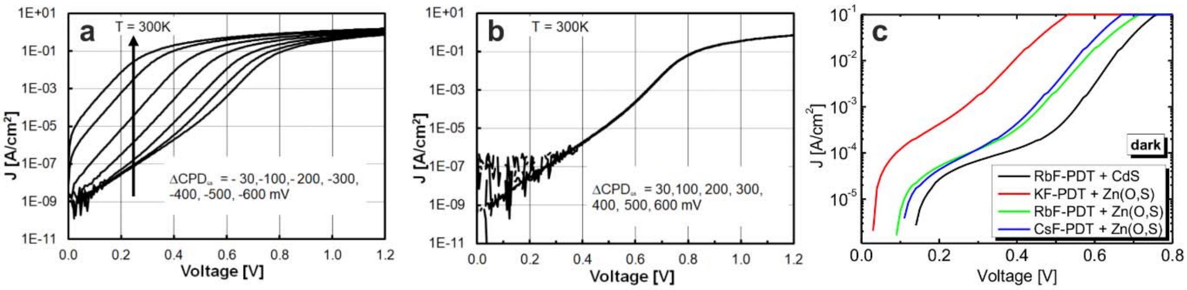

Supplementary Figure 5. Simulated dark J-V curves for different **a** downwards and **b** upwards band bendings (ΔCPD_{GB}) at the GB. **c** Experimental dark *J-V* curves at *T*=300 K for the three AlkF-PDT samples with a CBD-Zn(O,S) buffer layer and a RbF-PDT reference sample with CBD-CdS buffer layer. Comparison to the simulations in **a** indicates that the KF curve qualitatively agrees with a downwards band bending at GBs.

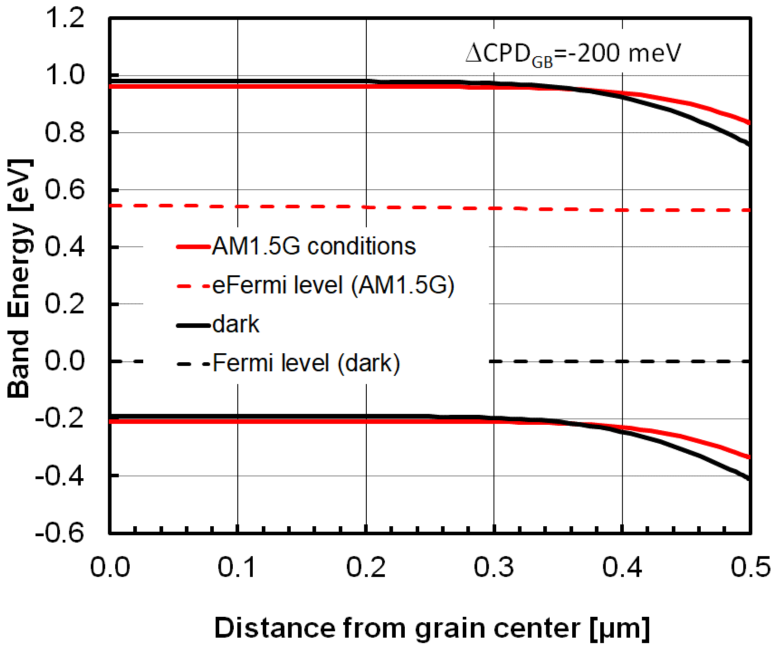

Supplementary Figure 6. Simulated GB band bending in the case of donor defects with a Gaussian energy distribution centered at mid-gap. The GB is at 0.5 μm from the grain center. The 200 meV band bending in the dark (black lines) is reduced to about 130 meV upon AM1.5G illumination (red lines), due to photo-generated electrons partially filling the donor levels. The applied bias is zero both in the dark (equilibrium condition) and under illumination (short-circuit condition).

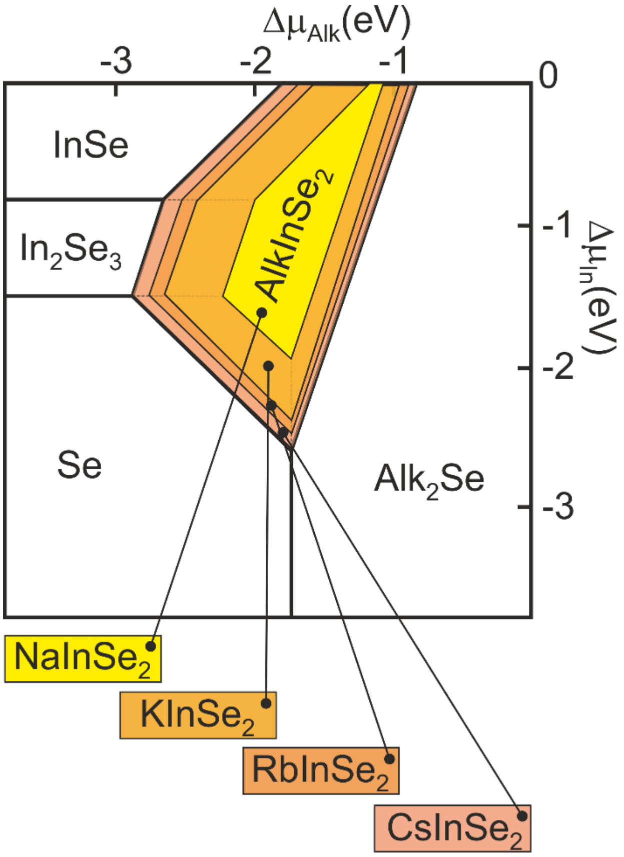

Supplementary Figure 7. Calculated Alk-In-Se stability diagrams for Alk = Na, K, Rb, and Cs. An increasing stability range is seen from Na → Cs. Data taken from Ref. [1]

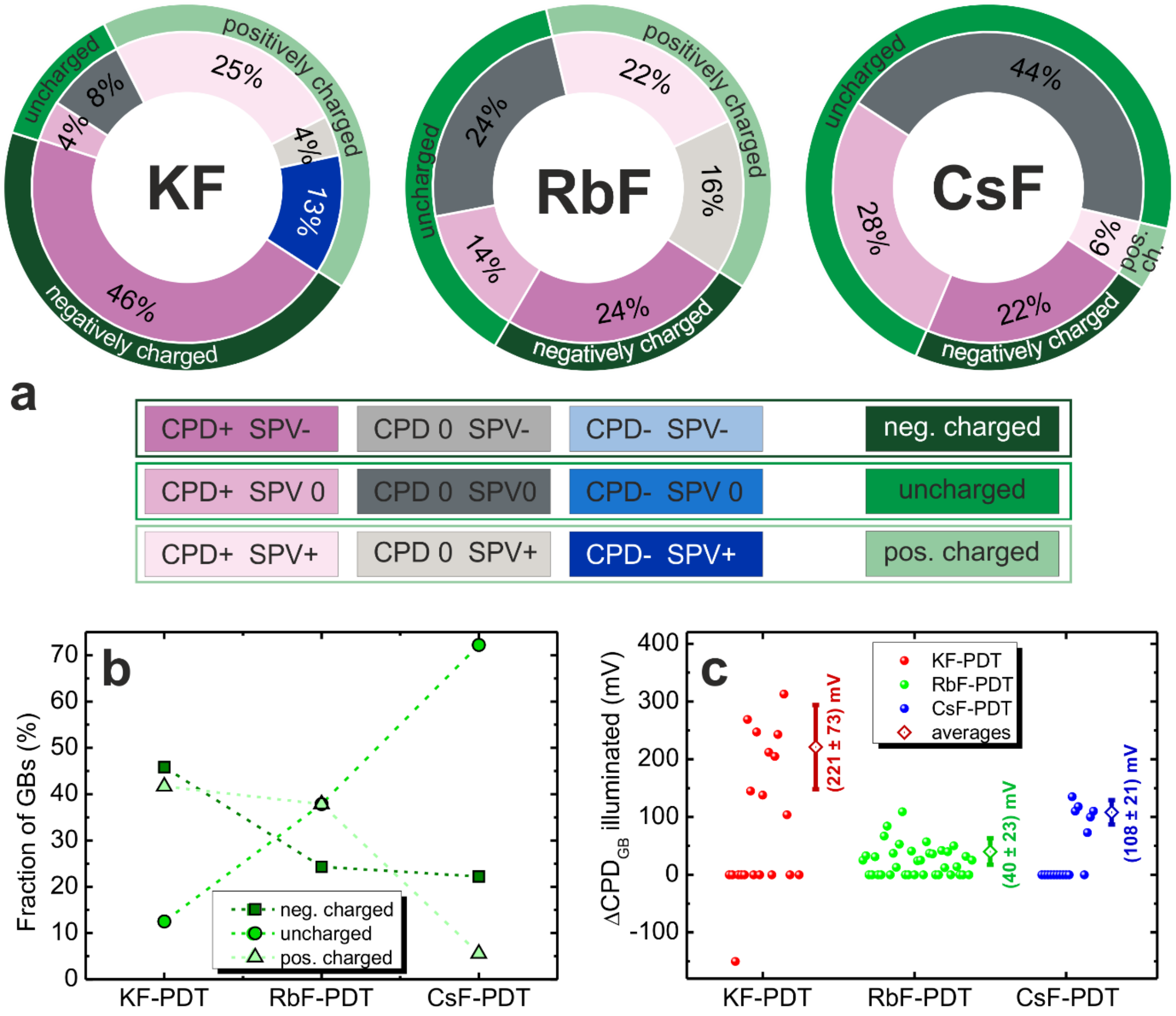

Supplementary Figure 8. Analysis of the CPD and SPV (difference between illuminated and dark KPFM measurements) at GBs on CIGSe absorbers with different AlkF-PDTs (annealed and rinsed after the PDT). **a** Pie charts representing the percentage of the combination of CPD and SPV as indicated in the legend, where from the left to right the data correspond to KF-, RbF-, and CsF-PDT. GBs with positive SPV are positively charged and those with negative SPV are negatively charged. **b** Dependence of the relative distribution of positive, negative, and uncharged GBs for the three AlkF-PDTs, indicating an increase of the fraction of uncharged GBs with increasing alkali mass. **c** Statistical evaluation of the CPD change at GBs under illumination ($\Delta CPD_{GB,light}$) for the three AlkF-PDT samples. The $\Delta CPD_{GB,light}$ under illumination should be related to the band offset, as photogenerated charges screen the fixed charges at the charged GBs reducing the related band bending contribution. The open diamonds indicate the average band offset with the standard deviation indicated by the error bars.

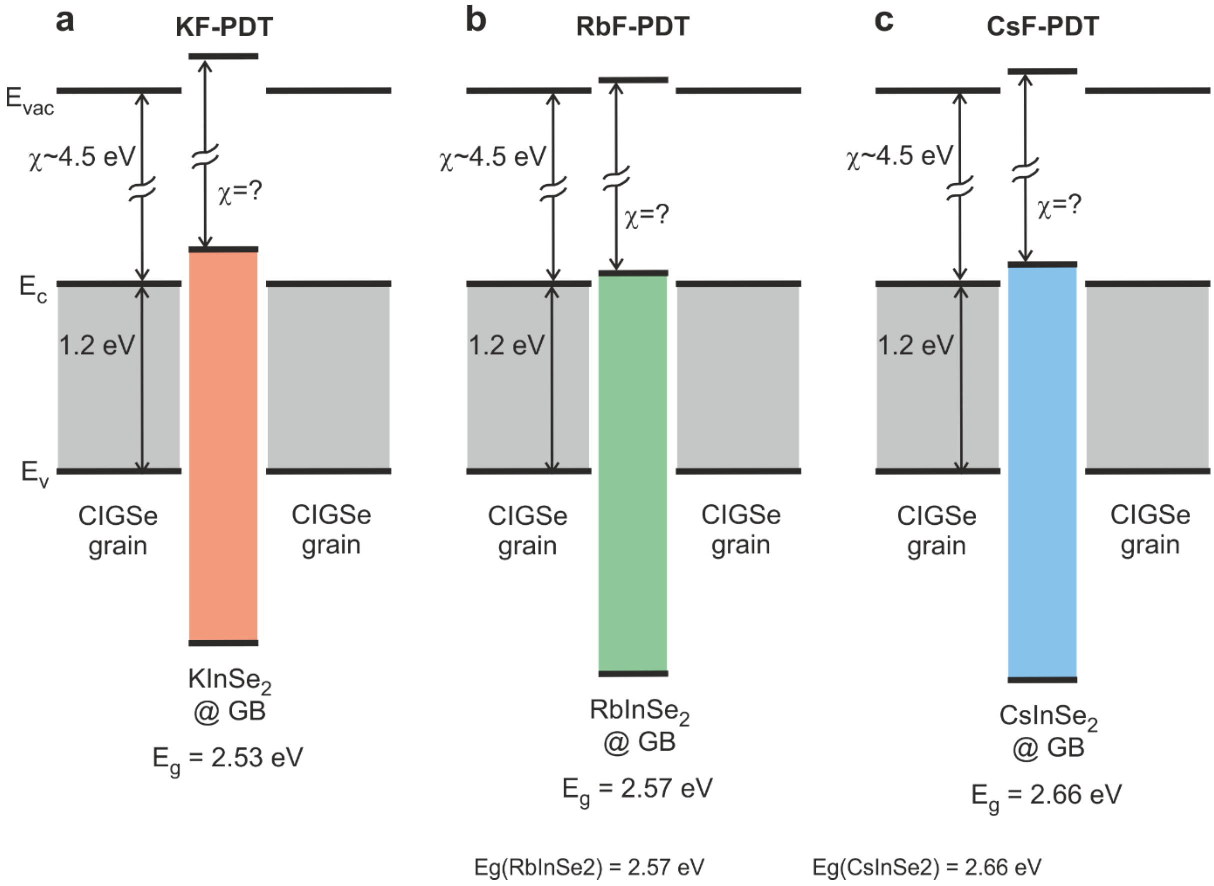

Supplementary Figure 9. Schematic band alignment for $AlkInSe_2$ phases at the grain boundaries of $Cu(In,Ga)Se_2$. The conduction band offset is estimated according to the results from the ΔCPD_{GB} values measured under illumination, as shown in Supplementary Figure 8c, and assuming that the electron affinity of $AlkInSe_2$ is similar to that of CIGSe. The band gap values indicated below the GB for the various $AlkInSe_2$ phases are taken from [1]. Note that a different electron affinity (χ) for the $AlkInSe_2$ would also modify the conduction band offset, as the ΔCPD_{GB} values are based on the KPFM measurement, which measures the distance from Fermi-level to the local vacuum level (E_{vac}). We do not specify the electron affinity values for the GB phase, as the presence of a continuous $AlkInSe_2$ phase at the GB is uncertain. (E_c – conduction band edge, E_v – valence band edge)

Supplementary Table 1. Values of all parameters used in the device simulations.

	MgF_2	ZnO:Al	i-ZnO	CdS	CIGSe
**Thickness [μm]**	0.105	0.2	0.08	0.03	3
**E_g [eV]**	-	3.3	3.3	2.4	Graded
**χ [eV]**	-	4.5	4.5	4.3	4.6
**Doping density $[cm^{-3}]$**	-	$4\cdot10^{19}$ (donor)	$1\cdot10^{17}$ (donor)	$1\cdot10^{17}$ (donor)	$1\cdot10^{16}$ (acceptor)
**Bulk trap concentration $[cm^{-3}]$**	-	$1\cdot10^{16}$ (mid-gap acceptor)	$1\cdot10^{16}$ (mid-gap acceptor)	$3\cdot10^{15}$ (mid-gap acceptor)	$6.67\cdot10^{14}$ (mid-gap donor)
**Bulk trap cross-sections $[cm^2]$**	-	$1\cdot10^{-12}$ (h) $1\cdot10^{-15}$ (e)	$1\cdot10^{-12}$ (h) $1\cdot10^{-15}$ (e)	$1\cdot10^{-12}$ (h) $1\cdot10^{-15}$ (e)	$1\cdot10^{-15}$ (h) $1\cdot10^{-15}$ (e)
**N_C $[cm^{-3}]$**	-	$2.3\cdot10^{18}$	$2.3\cdot10^{18}$	$2.3\cdot10^{18}$	$6.7\cdot10^{17}$
**N_V $[cm^{-3}]$**	-	$3.3\cdot10^{19}$	$3.3\cdot10^{19}$	$1.8\cdot10^{19}$	$1.5\cdot10^{19}$
**μ_e $[cm^2\ V^{-1}s^{-1}]$**		100	100	100	100
**μ_h $[cm^2\ V^{-1}s^{-1}]$**		25	25	25	25

Supplementary Table 2. Overview of literature results determining the concentration of alkali elements at grain boundaries. Abbreviations: APT – Atom probe tomography, TEM – Transmission electron microscopy, SLG – Soda lime glass, PDT – post-deposition treatment, 3D-TOF-SIMS – Three-dimensional time-of-flight secondary ion mass spectrometry, nano-XRF – nano X-ray fluorescence analysis.

Alkali element	Concentration	Method	Alkali source	Reference
K	0.5 – 1.9 at%	APT	SLG	[2]
Na<br>K	1.7 at%<br>0.2 at%	Transmission Kikuchi Diffraction and APT	SLG	[3]
Na<br>K	0.5 at%<br>1.35 at%	APT	SLG	[4]
Na<br>K	3.7 at%<br>0.3 at%	APT	SLG	[5]
Na<br>K	3.5 at%<br>0.3 at%	APT	SLG	[6]
Na<br>K	1.0-2.0 at%<br>0.3 at%	APT	SLG	[7]
Na	1.0-1.2 at%	APT	SLG	[8]
Na	0.5 at%	APT	SLG	[9]
Na<br>K	1.7 at%<br>0.035 at%		SLG	[10]
Na<br>K	0.3-0.4 at%<br>0.3-0.4 at%	APT	SLG	[11]
Na<br>K<br>Rb	0.4 at%<br>0.4 at%<br>< 1.5 at%	APT	RbF-PDT	[12]
Na<br>K<br>Rb	0.1-0.4 at%<br>0.1-0.9 at%<br>0.4-1.6 at%	APT and TEM	RbF-PDT	[13]
Rb	4.6 at%	3D-TOF-SIMS	RbF-PDT	[14]
Rb	< 24 at%	nano-XRF	RbF-PDT	[15]

Supplementary Note 1. Impact of AlkF-PDT on the CIGSe surface electronic properties

Between 20 and 50 measurements of topography and CPD for each sample with the three different AlkF-PDT were taken at different sample locations, separated by several hundred µm. This approach ensures a reliable assessment of the surface potentials. The CPD maps were converted to histograms where the potential distribution was fit with Gaussian curves, as shown in Fig. 1g of the main manuscript. From the fits, the peak maximum and the spread of the potential at 1/e of the maximum of the counts were extracted and an average value was determined for all inspected areas. The same procedure was applied for the CPD data obtained on alkali-treated CIGSe with an additional thin CBD-Zn(O,S) layer (i.e., 8 min deposition time). To convert the relative CPD values (CPD = Φ_{sample} - Φ_{tip}) to work function values, measurements on a reference Au sample have been taken repeatedly during the KPFM study. On the other hand, this procedure ensures that the work function of the Pt/Ir tip remains largely constant during the measurements. The statistical analysis of the surface potential for the CIGSe surfaces treated with different alkali fluorides is shown in Supplementary Figure 1.

The CIGSe samples with KF- and CsF-PDT show two distinct groups of work function values, separated by about 400 meV. We focus first on the higher work function values. The average work function values of the three AlkF-PDT CIGSe samples show differences between the three alkali elements with a maximum difference of 260 meV ($\Phi_{KF\text{-}PDT}$ = 5.20 eV, $\Phi_{RbF\text{-}PDT}$ = 5.46 eV, $\Phi_{CsF\text{-}PDT}$ = 5.24 eV); this value is in a similar range as the work function difference between the pure alkali metals K and Cs, which is ~340 meV. The lower work functions are rather similar for KF- and CsF-PDT CIGSe at $\Phi \approx 4.75$ eV. We attribute these regions with low work function on the KF- and CsF-treated samples to areas where the rinsing might not have fully washed the alkali-containing surface layer. We note that the ~400 mV difference between the low and the high work function groups cannot be explained by a tip modification, as frequent control measurements on the Au reference sample do confirm a constant CPD value of the tip.

The small differences in the work function value between the three different AlkF-PDTs can be analyzed further. Supplementary Figure 2 shows a slight correlation between the work function values and the open-circuit voltage of respective devices (measured in the as-grown state). Typically, V_{oc} and FF values of CIGSe solar cells with CBD-Zn(O,S) can be significantly increased by light-soaking and/or post-annealing procedures. The correlation between work function and V_{oc} might indicate that the observed change in work function might be at least partially attributed to a change in the Fermi-level position, reflecting an increased charge carrier concentration of the CIGSe absorber with heavier alkali-fluoride PDTs. In fact, it has been observed previously that AlkF-PDT leads to increased doping of the CIGSe which enables increased V_{oc} through the larger possible quasi-Fermi level splitting [16,17]. However, most likely, the different work function values are strongly influenced by the formation of surface phases containing the different alkali elements as a result of the AlkF-PDT. In fact, for CIGSe with a KF-PDT, $KInSe_2$ and In_2Se_3 surface phases have been observed by photoemission spectroscopy studies, which also reported a band widening and changes in the conduction band edge [18,19]. For RbF-PDT samples the occurrence of similar surface phases is still under discussion [20,21,22]. Recent results from thermal admittance spectroscopy and temperature dependent current-voltage characteristics [23] could not find any clear effect of the different alkali elements used in the PDT process of CIGSe, in agreement with the rather small influence on the work function observed here.

Supplementary Note 2. Impact of AlkF-PDT on the surface electronic properties of CIGSe with a thin Zn(O,S) buffer layer

The results for the identical CIGSe samples with an additional thin Zn(O,S) layer from an 8 min CBD process are shown on the right side in Supplementary Figure 1. The layer thickness of Zn(O,S) after a CBD of 8 min can be estimated to approximately 3 nm. For all AlkF-PDTs, the Zn(O,S) deposition changes the work function, leading to a similar increase of (210 ± 30) meV. The statistical analysis of the different areas on the sample shows that with the Zn(O,S) layer the spread of the work function values is larger for KF- and CsF-PDT samples and rather small for the RbF-PDT CIGSe. This more homogeneous value for the RbF-PDT is likely related to the fact that the CIGSe growth was optimized for the RbF-PDT. A relation to the best performance of respective devices is thus plausible.

For CIGSe with KF- and RbF-PDT, a similar increase of the work function upon a very thin buffer layer deposition (CdS) by CBD has previously been observed in studies of the early stages of the interface formation between alkali-PDT CIGSe and CdS [20,24]. The changes in the work function were attributed to a surface modification due to the complex chemical reaction which takes place in the chemical bath. It is believed that the CBD process induces continuous changes in the absorber surface, including the surface potential, until a sufficiently thick buffer layer is formed, which will then have a more homogenous and clear signature.

Supplementary Note 3. Impact of alkali-PDT on the local electronic properties at grain boundaries (GBs)

A detailed analysis of localized effects of AlkF-PDT at GBs was performed by analyzing a total of more than 50 images in 30 macroscopically different areas. Supplementary Figure 3 shows representative results on CIGSe samples with KF-, RbF-, and CsF-PDT, to illustrate the approach used for the GB analysis. The GBs are identified from the topography to ensure their selection independent of specific CPD changes, following an approach previously reported [24,25]. In some cases, where the identification of GBs is not straightforward, additional processed images (i.e. derivative or Laplacian filtered topography) are used. The surface potential at GBs is extracted from the CPD map by taking line profiles perpendicular to the GBs. An average CPD over a 100 to 200 nm wide and ~300 to 500 nm long segment is considered to reduce the noise level of the surface potential at the GB and the surrounding grain surfaces.

Supplementary Note 4. 3D device simulations considering the GB effects

The simulation of the solar cell – performed using the commercial Synopsys Sentaurus TCAD suite (https://www.synopsys.com/silicon/tcad.html) – is based on the solution of the Poisson, electron and hole continuity, and the stationary drift-diffusion equations including models for Shockley-Read-Hall (SRH) recombination.

The electrostatic potential Φ is the solution of the Poisson equation:

$$\nabla \cdot \left(\varepsilon \nabla \Phi + \vec{P}\right) = -q(p - n + N_D - N_A) - \rho_{trap} \ , \tag{1}$$

where ε is the electrical permittivity, $\vec{P}$ the ferroelectric polarization, q the elementary electronic charge, n and p the electron and hole densities, N_D and N_A the concentration of ionized donors and acceptors, respectively, and ρ_{trap} the charge density contributed by traps and fixed charges.

The electron and hole continuity equations are:

$$\nabla \cdot \vec{J}_n = q\left(R_{net,n} - G_{net,n}\right) + q\frac{\partial n}{\partial t} \tag{2}$$

$$-\nabla \cdot \vec{J}_p = q\left(R_{net,p} - G_{net,p}\right) + q\frac{\partial p}{\partial t} \ , \tag{3}$$

where $R_{net,n}$ and $R_{net,p}$ are the electron and hole net recombination rate, respectively, $G_{net,n}$ and $G_{net,p}$ are the electron and hole net generation rate, respectively, $\vec{J}_n$ and $\vec{J}_p$ are the electron and hole current density, respectively, and n and p are the electron and hole density, respectively.

Non-radiative recombination is described by the SRH model:

$$R_{net}^{SRH} = \frac{np - n_{i,eff}^2}{\tau_p(n+n_1) + \tau_n(p+p_1)} \ , \tag{4}$$

with

$$n_1 = n_{i,eff}\, exp\left(\frac{E_{trap}}{kT}\right) \tag{5}$$

$$p_1 = n_{i,eff}\, exp\left(\frac{-E_{trap}}{kT}\right) \ , \tag{6}$$

where E_{trap} is the difference between the defect level and the intrinsic level.

The cell is illuminated by the standard AM1.5G solar spectrum, and the light propagation through the layered media is calculated by the transfer matrix method (TMM). The optical coefficient for all materials were taken from [26]. The most relevant parameters used in the simulation are listed in Supplementary Table 1. It should be noted that the bandgap values in the table are the electronic bandgap values determining band offsets and transport behavior; the optical bandgap values are implicitly determined by the measured optical coefficients, which are inputs to the simulations. In particular, in the case of ZnO:Al the estimated Burnstein-Moss shift is 0.31 eV as compared to non-intentionally doped ZnO [26].

Since the physical and electrical characteristics of GB defects (acceptor or donor type, energy distribution, density, capture cross-sections) are unknown, and we aimed at qualitative indications about the effect of downward (i.e., hole barrier) or upward (i.e., electron barrier) GB band bending on the cell performance, we tested several values of GB band bending by decorating the GB with either *fixed* positive (downward band bending) or negative (upward band bending) charge of varying density.

In the simulations, ΔCPD_{GB} values are extracted from the band diagrams of the cell at equilibrium: in particular, ΔCPD_{GB} is measured as the band bending between the grain center and the grain boundary

($\Delta CPD_{GB} > 0$ means upward band bending, $\Delta CPD_{GB} < 0$ downward band bending). Once the ΔCPD_{GB} corresponding to a particular fixed charge concentration in the grain boundary is evaluated as explained, the cell is illuminated by the standard AM1.5G spectrum and the corresponding figures of merit are calculated.

In the presence of negative ΔCPD_{GB} (i.e., downward band bending at the GB), the GB gets richer and richer of electrons as ΔCPD_{GB} increases, as shown in Supplementary Figure 4 by the electron current density maps corresponding to the cases of ΔCPD_{GB} = -30 mV and -600 mV.

In fact, the band bending determines the formation of an electron channel that tends to shunt the junction, as indicated by the dark *J-V* curves of Supplementary Figure 5a. V_{oc} and FF are thus heavily degraded for large negative ΔCPD_{GB}. An upwards band bending, instead, causes the holes to accumulate around the GB, which does not alter the diode behavior (Supplementary Figure 5b).

Quantitatively different simulated behavior is expected in the case where the fixed GB charge is replaced with either donor (downward band bending) or acceptor (upward band bending) traps. For example, the same equilibrium downward band bending ΔCPD_{GB} = -200 mV can be obtained either with $1.25 \cdot 10^{11}$ cm^{-2} fixed positive charges, or with $1.28 \cdot 10^{11}$ cm^{-2} donor defects with a Gaussian energy distribution centered at mid-gap with variance $\sigma^2 = 9 \cdot 10^{-4}$ eV^2. However, unlike the fixed charge, the charge stored in the donor defects is a function of the quasi-Fermi level position, and as such depends on bias and illumination: as an example, a ΔCPD_{GB} = -200 mV in the dark is reduced to approximately -130 mV under AM1.5G conditions, as shown in Supplementary Figure 6. The behavior seen in Fig. 4 is therefore distorted when fixed charge is replaced with electrically active defects. Generally speaking, defects (either donors or acceptors) with capture cross sections small enough (e.g., 10^{-20} cm^2) to give negligible non-radiative recombination at the GB will show qualitatively similar trends but smaller effects than those shown in Fig. 4, due to reduced band bending upon illumination (Supplementary Figure 6); on the other hand, defects with cross sections large enough (e.g., 10^{-16} cm^2) to result in significant non-radiative recombination at the GB show – unlike the fixed charges of Fig. 4 – degraded J_{sc} both for negative and positive ΔCPD_{GB}.

It should be pointed out, however, that for our samples no hard experimental data is available as far as the defect model parameters (density, defect type, energy distribution, cross sections) are concerned, so direct quantitative comparison with the fixed charge case is very difficult to establish. For this reason, the discussion in the paper is limited to the simplified case of fixed GB charge and its qualitative effects on the cell's performance.

Supplementary Note 5. Surface photovoltage (SPV) of grain boundaries

A total of 79 GBs have been analyzed for the annealed and rinsed alkali-treated (KF, RbF, and CsF) samples in KPFM measurements realized in dark and under illuminated conditions. The $\Delta CPD_{GB,dark}$ was extracted from KPFM images measured under dark conditions. Subsequently, the same area was measured under illumination (635 nm diode laser) to extract the respective $\Delta CPD_{GB,light}$ values. The SPV of each individual GB was then calculated from the difference, $SPV_{GB} = \Delta CPD_{GB,light} - \Delta CPD_{GB,dark}$, where the SPV_{GB} can be positive, zero, or negative. The statistical distribution of the GBs with a specific barrier (ΔCPD_{GB} positive, zero, or negative) in relation to the respective SPV_{GB} is shown in Supplementary Figure 8.

If the presence of charges at the GB is responsible for the observed ΔCPD_{GB}, photo-generated charges as a result of sample illumination will screen the fixed GB charges leading to a smaller ΔCPD_{GB} under illumination. Therefore, the presence of a non-zero SPV at a GB indicates a charged GB. On the other hand, $SPV_{GB} = 0$ indicates that any ΔCPD_{GB} observed at these GBs is possibly due to a different phase present at the GB.

Supplementary References

1. Malitckaya, M., Komsa, H.-P., Havu, V. & Puska, M. J. Effect of Alkali Metal Atom Doping on the $CuInSe_2$ -Based Solar Cell Absorber. *J. Phys. Chem. C* **121,** 15516–15528 (2017).
2. Stokes, A., Al-Jassim, M., Diercks, D., Clarke, A. & Gorman, B. Impact of Wide-Ranging Nanoscale Chemistry on Band Structure at $Cu(In,Ga)Se_2$ Grain Boundaries. *Sci. Rep.* **7,** 14163 (2017).
3. Schwarz, T. *et al.* Correlative transmission Kikuchi diffraction and atom probe tomography study of $Cu(In,Ga)Se_2$ grain boundaries. *Prog. Photovoltaics Res. Appl.* **26,** 196–204 (2018).
4. Cojocaru-Mirédin, O., Schwarz, T. & Abou-Ras, D. Assessment of elemental distributions at line and planar defects in $Cu(In,Ga)Se_2$ thin films by atom probe tomography. *Scr. Mater.* **148,** 106–114 (2018).
5. Abou-Ras, D. *et al.* Confined and chemically flexible grain boundaries in polycrystalline compound semiconductors. *Adv. Energy Mater.* **2,** 992–998 (2012).
6. Choi, P.-P. *et al.* Atom Probe Tomography of Compound Semiconductors for Photovoltaic and Light-Emitting Device Applications. *Micros. Today* **20,** 18–24 (2012).
7. Cojocaru-Mirédin, O. *et al.* Characterization of Grain Boundaries in $Cu(In,Ga)Se_2$ Films Using Atom-Probe Tomography. *IEEE J. Photovoltaics* **1,** 207–212 (2011).
8. Couzinie-Devy, F., Cadel, E., Barreau, N., Arzel, L. & Pareige, P. Atom probe study of Cu-poor to Cu-rich transition during $Cu(In,Ga)Se_2$ growth. *Appl. Phys. Lett.* **99,** 232108 (2011).
9. Couzinie-Devy, F., Cadel, E., Barreau, N., Arzel, L. & Pareige, P. Na distribution in $Cu(In,Ga)Se_2$ thin films: Investigation by atom probe tomography. *Scr. Mater.* **104,** 83–86 (2015).
10. Cojocaru-Mirédin, O. *et al.* Atom Probe Tomography Studies on the $Cu(In,Ga)Se_2$ Grain Boundaries. *J. Vis. Exp.* **74**, e50376 (2013).
11. Stokes, A., Al-Jassim, M., Diercks, D. & Gorman, B. Alkali segregation and matrix concentrations in thin film $Cu(In,Ga)Se_2$ at targeted interfaces characterized in 3-D at the nanoscale. *IEEE 42nd Photovolt. Spec. Conf. PVSC 2015* 1–4 (2015).
12. Vilalta-Clemente, A. *et al.* Rubidium distribution at atomic scale in high efficient $Cu(In,Ga)Se_2$ thin-film solar cells. *Appl. Phys. Lett.* **112,** 103105 (2018).
13. Raghuwanshi, M. *et al.* Influence of RbF post deposition treatment on heterojunction and grain boundaries in high efficient (21.1%) $Cu(In,Ga)Se_2$ solar cells. *Nano Energy* **60,** 103–110 (2019).
14. Wuerz, R., Hempel, W. & Jackson, P. Diffusion of Rb in polycrystalline $Cu(In,Ga)Se_2$ layers and effect of Rb on solar cell parameters of $Cu(In,Ga)Se_2$ thin-film solar cells. *J. Appl. Phys.* **124,** 165305 (2018).
15. Schöppe, P. *et al.* Overall Distribution of Rubidium in Highly Efficient $Cu(In,Ga)Se_2$ Solar Cells. *ACS Appl. Mater. Interfaces* **10,** 40592–40598 (2018).
16. Pianezzi, F. *et al.* Unveiling the Effects of Post-Deposition Treatment with Different Alkaline Elements on the Electronic Properties of CIGS Thin Film Solar Cells. *Phys. Chem. Chem. Phys.* **16,** 8843–8851 (2014).
17. Laemmle, A., Wuerz, R. & Powalla, M. Efficiency Enhancement of $Cu(In,Ga)Se_2$ Thin-Film Solar Cells by a Post-Deposition Treatment with Potassium Fluoride. *Phys. Status Solidi - Rapid Res. Lett.* **7,** 631–634 (2013).
18. Handick, E. *et al.* Potassium Postdeposition Treatment-Induced Band Gap Widening at $Cu(In,Ga)Se_2$ Surfaces - Reason for Performance Leap? *ACS Appl. Mater. Interfaces* **7,** 27414–27420 (2015).
19. Handick, E. *et al.* Formation of a K-In-Se Surface Species by NaF/KF Postdeposition Treatment of $Cu(In,Ga)Se_2$ Thin-Film Solar Cell Absorbers. *ACS Appl. Mater. Interfaces* **9,** 3581–3589

(2017).

20. Nicoara, N. *et al.* Evidence for Chemical and Electronic Nonuniformities in the Formation of the Interface of RbF-Treated Cu(In,Ga)Se_2 with CdS. *ACS Appl. Mater. Interfaces* **9,** 44173–44180 (2017).

21. Ishizuka, S. *et al.* Group III Elemental Composition Dependence of RbF Postdeposition Treatment Effects on Cu(In,Ga)Se_2 Thin Films and Solar Cells. *J. Phys. Chem. C* **122,** 3809−3817 (2018).

22. Hauschild, D. *et al.* Impact of a RbF Postdeposition Treatment on the Electronic Structure of the CdS/Cu(In,Ga)Se_2 Heterojunction in High-Efficiency Thin-Film Solar Cells. *ACS Energy Lett.* **2,** 2383–2387 (2017).

23. Werner, F. *et al.* Alkali treatments of Cu(In,Ga)Se_2 thin-film absorbers and their impact on transport barriers. *Prog. Photovoltaics Res. Appl.* **26,** 911–923 (2018).

24. Nicoara, N. *et al.* Effect of the KF Post-Deposition Treatment on Grain Boundary Properties in Cu(In, Ga)Se_2 Thin Films. *Sci. Rep.* **7,** 41361–41367 (2017).

25. Baier, R. *et al.* Electronic properties of grain boundaries in Cu(In,Ga)Se_2 thin films with various Ga-contents. *Sol. Energy Mater. Sol. Cells* **103,** 86–92 (2012).

26. Carron, R. *et al.* Refractive indices of layers and optical simulations of Cu(In,Ga)Se_2 solar cells. *Sci. Technol. Adv. Mater.* **19,** 396–410 (2018).
