# Supplementary material for: Direct evidence for grain boundary passivation in Cu(In,Ga)Se2 solar cells through alkali-fluoride post-deposition treatments
Source: Nat Commun. 2019 Sep 4;10:3980. doi: 10.1038/s41467-019-11996-y (PMC6726603; doi:10.1038/s41467-019-11996-y)
Supplement: Supplementary file 2 — Solar Cells Reporting Summary [file 41467_2019_11996_MOESM2_ESM.pdf]

nature research

Corresponding author(s): Sascha Sadewasser

Solar Cells Reporting Summary

Nature Research wishes to improve the reproducibility of the work that we publish. This form is intended for publication with all accepted papers reporting the characterization of photovoltaic devices and provides structure for consistency and transparency in reporting. Some list items might not apply to an individual manuscript, but all fields must be completed for clarity.

For further information on Nature Research policies, including our data availability policy, see Authors & Referees.

Experimental design

Please check: are the following details reported in the manuscript?

1. Dimensions

Item	Reported	Details
Area of the tested solar cells	☒ Yes ☐ No	0.5 cm2 total area, stated in Methods section
Method used to determine the device area	☐ Yes ☒ No	simple measurement possible due to large area; cell area defined through patterning

2. Current-voltage characterization

Item	Reported	Details
Current density-voltage (J-V) plots in both forward and backward direction	☐ Yes ☒ No	Not relevant for CIGSe materials: no hysteresis effects have been reported
Voltage scan conditions<br>*For instance: scan direction, speed, dwell times*	☐ Yes ☒ No	forward scan direction; speed and dwell times not relevant for CIGSe materials
Test environment<br>*For instance: characterization temperature, in air or in glove box*	☐ Yes ☒ No	Standard testing conditions; in air
Protocol for preconditioning of the device before its characterization	☒ Yes ☐ No	15 min cold light soaking is stated in the Methods section
Stability of the J-V characteristic<br>*Verified with time evolution of the maximum power point or with the photocurrent at maximum power point; see ref. 7 for details.*	☐ Yes ☒ No	Not relevant for CIGSe materials

3. Hysteresis or any other unusual behaviour

Item	Reported	Details
Description of the unusual behaviour observed during the characterization	☐ Yes ☒ No	Not relevant for CIGSe materials
Related experimental data	☐ Yes ☒ No	Not relevant for CIGSe materials

4. Efficiency

Item	Reported	Details
External quantum efficiency (EQE) or incident photons to current efficiency (IPCE)	☐ Yes ☒ No	not relevant for present results and interpretation
A comparison between the integrated response under the standard reference spectrum and the response measure under the simulator	☐ Yes ☒ No	not applicable
For tandem solar cells, the bias illumination and bias voltage used for each subcell	☐ Yes ☒ No	not applicable

5. Calibration

Item	Reported	Details
Light source and reference cell or sensor used for the characterization	☐ Yes ☒ No	AAA solar simulator; xenon lamp, AM1.5G spectrum; Si reference cell
Confirmation that the reference cell was calibrated and certified	☐ Yes ☒ No	standard test conditions

Calculation of spectral mismatch between the reference cell and the devices under test

- [ ] Yes
- [x] No

not applicable

6. Mask/aperture

Size of the mask/aperture used during testing

- [ ] Yes
- [x] No

not applicable

Variation of the measured short-circuit current density with the mask/aperture area

- [ ] Yes
- [x] No

not applicable

7. Performance certification

Identity of the independent certification laboratory that confirmed the photovoltaic performance

- [ ] Yes
- [x] No

not applicable

A copy of any certificate(s)
Provide in Supplementary Information

- [ ] Yes
- [x] No

not applicable

8. Statistics

Number of solar cells tested

- [ ] Yes
- [x] No

only reference solar cell measurements stated

Statistical analysis of the device performance

- [ ] Yes
- [x] No

only reference solar cell measurements stated

9. Long-term stability analysis

Type of analysis, bias conditions and environmental conditions
For instance: illumination type, temperature, atmosphere humidity, encapsulation method, preconditioning temperature

- [ ] Yes
- [x] No

not applicable
